# The influence of the social and cultural environment on maternal mortality in Nigeria: Evidence from the 2013 demographic and health survey

**DOI:** 10.1371/journal.pone.0190285

**Published:** 2017-12-29

**Authors:** Oluwatosin Ariyo, Ifeoma D. Ozodiegwu, Henry V. Doctor

**Affiliations:** 1 Department of Community and Behavioral Health, East Tennessee State University, Johnson City, Tennessee, United States of America; 2 Department of Epidemiology and Biostatistics, East Tennessee State University, Johnson City, Tennessee, United States of America; 3 Department of Information, Evidence and Research, World Health Organization, Regional Office for the Eastern Mediterranean, Cairo, Egypt; University of Miami, UNITED STATES

## Abstract

**Introduction:**

Reducing maternal mortality remains a priority for global health. One in five maternal deaths, globally, are from Nigeria.

**Objective:**

This study aimed to assess the sociocultural correlates of maternal mortality in Nigeria.

**Methods:**

We conducted a retrospective analysis of nationally representative data from the 2013 Nigeria Demographic and Health Survey. The analysis was based on responses from the core women’s questionnaire. Maternal mortality was categorized as ‘yes’ for any death while pregnant, during delivery or two months after delivery (as reported by the sibling), and ‘no’ for deaths of other or unknown causes. Multilevel logistic regression analysis was conducted to test for association between maternal mortality and predictor variables of sociocultural status (educational attainment, community women’s education, region, type of residence, religion, and women’s empowerment).

**Results:**

Region, Religion, and the level of community women’s education were independently associated with maternal mortality. Women in the North West were more than twice as likely to report maternal mortality (OR: 2.14; 95% CI: 1.42–3.23) compared to those in the North Central region. Muslim women were 52% more likely to report maternal deaths (OR: 1.52; 95% CI: 1.10–2.11) compared to Christian women. Respondents living in communities where a significant proportion of women have at least secondary schooling were 33% less likely to report that their sisters died of pregnancy-related causes (OR: 0.67; 95% CI: 0.48–0.95).

**Conclusion:**

Efforts to reduce maternal mortality should implement tailored programs that address barriers to health-seeking behavior influenced by cultural beliefs and attitudes, and low educational attainment. Strategies to improve women’s agency should be at the core of these programs; they are essential for reducing maternal mortality and achieving sustainable development goals towards gender equality. Future studies should develop empirically evaluated measures which assess, and further investigate the association between women’s empowerment and maternal health status and outcomes.

## Introduction

Reducing maternal mortality remains a priority for global health [1]. Each day, about 830 women die from preventable pregnancy-related causes [2]. Over 50% of these deaths occur in Sub-Saharan African countries [2]. These estimates served as the basis for the development of the fifth Millennium Development Goals (MDG 5) to reduce maternal mortality ratio by 75% between 1990 and 2015. Although, 2015 global and regional estimates of maternal mortality indicate a downward trend, aggregate statistics mask differences in each country’s progress [3]. While some Sub-Saharan African countries such as Rwanda and Cape Verde met the MDG 5, estimates of maternal mortality in Nigeria indicates slow progress [3]. In 2015, Nigeria contributed to 1 in 5 and 1 in 4 global and regional maternal deaths, respectively [3]. Findings from the 2008 and 2013 Nigeria Demographic and Health Survey (DHS) revealed an increase in maternal mortality ratio (MMR) from 545 to 576 deaths per 100,000 live births [4]. Based on these estimates, a Nigerian woman in her reproductive years has a 1 in 22 lifetime risk of maternal death, compared to the average lifetime risk of 1 in 4900 and 1 in 180 for women in high and low-income countries, respectively [2,5]

The existence of inadequate antenatal, intrapartum and postpartum care are major risk factors for maternal mortality for Nigerian women [6–10]. Existing interventions to reduce maternal mortality in Nigeria have centered on proximate determinants such as health-seeking behavior and access to services, with little focus on the social and cultural factors that influence maternal mortality [11]. Evidence suggests that the most effective programs are those that address the community context as well as the clinical setting [6,12]. Thus, despite the implementation of several Safe Motherhood Initiatives to provide health services to pregnant women, mortality rates have not significantly decreased [2].

Nigerian women, compared to men, experience inequality in education, income and social standing; these factors affect their health by creating inequitable conditions. Conceptual models which predict the risk of maternal mortality indicate that women’s status in their families and communities, as evidenced by their social and legal autonomy, education, income, and occupation, has implications for their access to health services, health-seeking behavior, reproductive status, and health status [13]. Several studies highlight women’s socioeconomic status as predictors of poor maternal health [9,10,14]. A recent study showed that many poor and uneducated women in Nigeria do not deliver at health facilities or in the presence of a skilled birth attendant [9]. Another study which investigated the relationship between women’s social status and receipt of adequate antenatal and intrapartum care showed that participating in household decision making among women was associated with an increased likelihood of receiving antenatal care, delivering in a health facility and delivery in the presence of a skilled birth attendant [10]. This study also showed that a strong justification of wife beating among Nigerian women was linked to a decreased likelihood of receiving antenatal care [10].

Efforts to meet the Sustainable Development Goal 3 to bring MMR below 70 deaths per 100,000 live births is hardly attainable without a review of current strategies and the identification of a wider range of determinants which consider the socioecological context in which Nigerian women live. The purpose of this study was to investigate the association between social and cultural factors and maternal mortality. We conducted this retrospective analysis based on the hypothesis that indices of low socioeconomic status and gender inequality are associated with maternal mortality.

## Conceptual framework

The conceptual underpinnings for this study is based on the McCarthy framework of determinants of maternal mortality [13]. This framework elicits the pathway through which a woman’s socioeconomic status and her social and cultural environment influence her health and reproductive status, access to and utilization of health services across her life-course, and her health behaviors and thus determine her risk for maternal mortality.

We adapt this framework to our study and posit that indicators of socioeconomic status (wealth index, education, spousal educational differences, community women’s education, type of residence), women empowerment (autonomy, economic empowerment and attitudes towards domestic violence) and culture (region of residence and religion) are independently associated with maternal mortality at a distal level [9,10,12–15]. We hypothesize that their independent effects will be confounded by a variety of factors at the proximal level.

Due to the paucity of variables in the dataset, at the proximal level, we only selected age at death as a confounder. In our dataset, age at death was independently associated with pregnancy-related mortality and associated with other exposure variables. The relationship with the outcome is in line with an aggregate study using the DHS which indicates that older women in the 20–34 age group have a higher likelihood of maternal mortality [16]. Hence, if sociocultural factors are unequally distributed among our study population, not adjusting for effect of age could potentially lead to erroneous estimates. We test these hypotheses using our statistical analysis.

## Materials and methods

This study used nationally-representative data from the 2013 Nigeria Demographic and Health Survey (DHS) which contains information on population and health estimates at the national, state and zonal level [4]. Using a household listing, eligible women aged 15–49 years were selected for the women’s questionnaire. A total of 38,948 interviews were completed with a response rate of 97.6% [4]. Data for pregnancy-related deaths were obtained from the women’s questionnaire. The direct sisterhood method based on sibling history was used to estimate pregnancy-related deaths [1,3,17]. The direct sisterhood method is widely used to estimate pregnancy related maternal mortality rates in large population surveys because it produces nationally representative data, is easily included in multipurpose surveys and it generates a current estimate [18–20]. The direct sisterhood method has been found to produce similar estimates with the verbal autopsy method in Zambia [21]. Each respondent was asked about the name, age, sex, survival status, and age/year of death of each sibling born to the same mother [4,22]. If a dead sibling was a female of reproductive age, additional questions were asked about the period of their death relative to a pregnancy [22]. This analysis was exempted from ethical approval by the Eastern Tennessee State University Institutional Review Board because it was a secondary analysis of publicly available data.

A description of the selected measures in the study and how the characteristics were measured using the direct sisterhood method is presented in Table 1. Except for the wealth index variable, the table provides the responses on which each measure is based. The wealth index variable is an indicator of a household’s living standard constructed with Principal Component Analysis using data collected on ownership of specific assets (such as television and bicycle; materials used for housing construction; and sources of water and types of sanitation facilities) believed to be correlated with socioeconomic status. Additional details of the construction of the wealth index for the Nigeria 2013 DHS is provided elsewhere by the DHS program [23]. Reported deaths of siblings aged 12 years and older were categorized as: ‘maternal mortality’, if reported death occurred during pregnancy, delivery or two months after delivery and non-maternal mortality, for deaths not related to pregnancy. We estimated the predicted likelihood of pregnancy-related mortality at each level of the variable holding all other variables in the model at their means.

**Table 1:** Characteristics of pregnancy-related deaths identified using the direct sisterhood method, Nigeria Demographic and Health Survey 2013.

Measure	Categories used	Direct sisterhood method
Pregnancy-related deaths	• Pregnancy • Delivery • Post-partum	Reporting sister responded to the question: “Was [name] pregnant when she died?”; “Did [name] die during childbirth?”; “Did [name] die within two months after the end of a pregnancy or childbirth?”
**Distal Determinants**		
**Socioeconomic factors**		
Wealth index	• Poor • Middle • Rich”	The household asset index, calculated at the household level, is reported for the household to which the reporting sister belongs as proxy.
Residence	• Urban • Rural	Based on the sample allocation of reporting sister for each sampled area
Highest educational attainment	• No schooling • Primary • Secondary • Higher	Reporting sister is asked whether she has ever attended school, and if so, what were the highest level and class that she completed; educational attainment reported is that of the reporting sister as proxy.
Community women’s education	• Low • High • Medium	Community level education was measured as the proportion of women with at least a secondary education in the sampling cluster. The measure was categorized into three tertiles: low, medium and high [24].
Spousal educational difference	• Equal • Wife higher • Husband higher	Calculated as the difference in years of educational attainment between reporting sisters and their husbands as proxy.
**Women empowerment**		
Autonomy in decision making	• Autonomous (either respondent alone or with husband/partner) • Less autonomous (husband/partner/someone/others)	Based on responses to four questions: (1) Who usually decides how the money respondent earns will be used? (2) Who usually decides how the money husband earn will be used? (3) Who usually makes decisions about health care for yourself? (4) Who usually makes decisions about making major household purchases? From these four items, we created dichotomous responses—women who reported making the decisions by themselves or with their husbands or partners were treated as autonomous and assigned a value of 0 for autonomy and 1 otherwise. A similar approach has been used elsewhere [25,26].
Economic empowerment	• Doesn’t own; or owns alone/jointly only/both alone and jointly • The ownership is for land or a house	Based on reporting sister responses to two questions: (1) Do you own any land either alone or jointly with someone else? (2) Do you own this or any other house either alone or jointly with someone else?
Attitude towards domestic violence	• Yes • No • Don’t know	Based on responses to respondent’s opinion on whether a husband is justified in hitting or beating his wife in the following situations: (a) goes out; (b) neglects children; (c) argues with husband; (4) refuses sex; and (5) burns food.
**Culture**		
Region of residence	• North Central • North East • North West • South East • South-South • South West	Based on the sample allocation of reporting sister for each sampled area as categorized by census distribution [4]
Religion	• Christianity • Islam • Other	Reporting sister is asked, “What is your religion”. With this question, 3 main categories are created. “Traditional” and “Other” religions are combined into “Other” due to few categories.
**Proximal Determinants**		
Age at death	• 12–19; 20–29; 30–39; 40–49	Reporting sister responded to the question: “How old was [name] when she died?”

### Statistical analysis

Statistical analyses were conducted via survey procedures including weights to account for the multistage survey design used in the DHS. The distributions among women with respect to the occurrence of pregnancy-related mortality and sociodemographic characteristics were compared using chi-square contingency tables and applying weighted frequencies. Survey weights were adjusted to overcome survivor bias by multiplying the weight by the inverse of the number of surviving female siblings of reproductive age [27].Univariate tests were used to summarize and identify patterns in the data focusing on the selected variable as described in Table 1. To investigate an association between pregnancy-related mortality and multiple predictor variables we used multilevel logistic regression model to estimate the magnitude of association in form of odds ratios (ORs) between pregnancy-related mortality and selected predictor variables. In particular, multilevel models were constructed using the mixed effects modeling procedure in Stata 14 to account for the hierarchical structure of the Nigeria DHS where data were collected in nested units [28].That is, women were clustered within households that were in turn clustered within the primary sampling unit. The regression model, therefore, consisted of two sub-models at the individual level (level 1) and the communities (level 2).

For the analysis, the explanatory factors were classified into two levels: individual- and community-level factors. Individual-level factors included: wealth index, highest education attainment, spousal educational difference, autonomy, economic empowerment, attitudes towards domestic violence, age at death, and religion. Community-level factors included: residence, region, and community women’s education. Three models containing variables of interest were fitted. **Model 0** (empty model) contained no exposure variable and only focused on the decomposition of total variance into its individual and community components. **Model 1** contained individual-level variables whereas **Model 2** included community-level variables as a full model.

The two-level multi-level model is written as follows, Eq (1):

$$logit\;(\pi_{ij})=\log\left(\frac{\pi_{ij}}{1-\pi_{ij}}\right)=\beta_{0}+X_{ij}+u_{0j}$$ (1)

where *π*_*ij*_ is the probability of dying for the *i*th woman in *j*th community, *X*_*ij*_ is a vector of covariates corresponding to the *i*th mother in the *j*th community, *β*_0_ is a vector of unknown parameters, *u*_0*j*_ is the random effect at the community level. The intercept or average probability of observing pregnancy-related mortality is assumed to vary randomly across women and communities. With this approach, the fixed effects (measures of association) are expressed as odds ratios (OR) with associated 95% confidence intervals (CI). Goodness of fit of the multilevel model was tested by the log likelihood ratio (LR) test.

Statistical significance was assessed at *P* < 0.05. Independent variables were subjected to multi-collinearity tests by performing correlations, variable inflation factor (VIF) and tolerance tests [29]. The mean VIF was 1.43 whereas tolerance values were at least 0.5. The tests indicated no cause for concern for collinearity. We also tested the model specification in Stata using post-estimation commands and results showed that conditional on the model specification the independent variables were specified correctly since the prediction squared variable had no explanatory power (β = 0.006, p = 0.616). We applied sample weights for descriptive analyses using the Stata ‘svy’ command to account for undercounting and overcounting due to the sample design of the survey. All statistical analyses were performed using Stata Version 14 [30].

## Results

### Proportion of pregnancy-related deaths

A total of 3,302 deaths were identified by women interviewed in the survey using the direct sisterhood method. Table 2 presents the proportion of pregnancy-related deaths identified by the direct sisterhood methods by selected characteristics of the reporting sister. Overall, the percent of pregnancy-related deaths was 33.4 (95% Confidence Interval (C.I): 30.7–36.1). The proportion of reported pregnancy-related was highest in the North West (53.6%). Among poor women, reported deaths were twice that of rich women (45.4% vs. 22.7%). Pregnancy-related deaths were also reported by almost half of participants that practiced Islam (46.2%) and those with no formal schooling (47.4%). Women with less autonomy had roughly twice the proportion of deaths seen in autonomous women (38.5% vs. 20.7%). The mean age at death for women with pregnancy-related mortality was 26.7 years (SD = 8.1).

**Table 2:** Proportion of pregnancy-related deaths by selected characteristics identified by the direct sisterhood method, Nigeria Demographic and Health Survey 2013 (n = 3,302).

Characteristics		Percent of deaths due to…	
		Non-maternal (n = 2,199)	Maternal (n = 1,103)
**Wealth index**	Poor	54.6	45.4
	Middle	69.3	30.7
	Rich	77.3	22.7
**Residence**	Urban	73.6	26.5
	Rural	61.6	38.4
**Community women’s education**	Low	50.8	49.2
	Medium	71.3	28.7
	Higher	79.3	20.7
**Highest educational** **attainment**	No schooling	52.7	47.4
	Primary	74.2	25.8
	Secondary	76.6	23.4
	Higher	78.5	21.6
**Spousal educational** **difference**	Equal	61.4	38.6
	Wife higher	74.4	25.6
	Husband/partner higher	69.3	30.7
**Autonomy**	Autonomous	79.3	20.7
	Less autonomous	61.5	38.5
**Economic empowerment**			
Owns land	No	65.8	34.2
	Yes	70.2	29.8
Owns house	No	65.8	34.2
	Yes	70.0	30.0
**Attitudes towards domestic violence**	Positive (No)	68.8	31.2
	Negative (Yes)	62.7	37.3
**Region**	North Central	66.8	33.2
	North East	65.2	34.8
	North West	46.5	53.6
	South East	75.2	24.9
	South South	84.2	15.8
	South West	79.5	20.5
**Religion**	Christianity	79.1	20.9
	Islam	53.8	46.2
	Other	62.6	37.4
**Age at death**	12–19	70.0	30,0
	20–29	61.5	38.5
	30–39	61.4	38.6
	40–49	78.1	21.9
	Mean age at death (SD)*^+^	26.6 (9.8)	26.7 (8.1)
**All deaths**		**66.6**	**33.4**

Notes

*SD = Standard deviation. Some percentages may not add up to 100 due to rounding of figures.

^+^Mean age at death for all women was 27.6 years (SD = 10.5).

### Socio-demographic characteristics of deceased women

Table 3 presents the individual characteristics of the deceased women, with the reporting sister characteristics used as proxy in the direct sisterhood method. The distribution of all variables except for whether the sister owned land or a house and category of death was significant at *P* < 0.05. For each category of socio-demographic characteristics, reporting sisters were mostly living in poor households (35.9%); from North West region (26.2%); practicing Christianity (50.4%); living in rural areas (58.4%); with no formal schooling (40.2%); with similar years of schooling as the husband/partner (59.8%);with less autonomy (78.5%); did not own land nor a house (almost 80%, each); and 38.7% justified husband/partner beating a wife under certain circumstances reported a higher number of deaths.

**Table 3:** Individual characteristics among pregnancy-related deaths identified by the direct sisterhood method, Nigeria Demographic and Health Survey 2013.

Characteristics		Reported maternal deaths		All women (n = 3,302) N (%)	*P*-value
		No (n = 2,199) N (%)	Yes (n = 1,103) N (%)		
**Wealth index**					0.0000
	Poor	646 (29.4)	538 (48.8)	1,185 (35.9)	
	Middle	737 (33.5)	327 (29.6)	1,064 (32.2)	
	Rich	815 (37.1)	239 (21.6)	1,053 (31.9)	
**Residence**					0.0000
	Urban	1,010 (45.9)	363 (32.9)	1,373 (41.6)	
	Rural	1,189 (54.1)	740 (67.1)	1,929 (58.4)	
**Community women’s education**					0.0000
	Low	567 (27.7)	502 (53.5)	1,069 (36.3)	
	Medium	819 (31.9)	311 (25.5)	1,130 (29.8)	
	Higher	884 (40.4)	219 (21.0)	1,103 (33.9)	
**Highest educational** **attainment**					0.0000
	No schooling	699 (31.8)	628 (57.0)	1,327 (40.2)	
	Primary	547 (24.9)	190 (17.2)	737 (22.3)	
	Secondary	739 (33.6)	226 (20.5)	965 (29.2)	
	Higher	214 (9.7)	59 (5.3)	273 (8.3)	
**Spousal educational** **difference**					0.0007
	Equal	1,006 (56.1)	632 (66.1)	1,638 (59.6)	
	Wife higher	256 (14.3)	88 (9.2)	344 (12.5)	
	Husband/partner higher	531 (29.6)	235 (24.6)	767 (27.9)	
**Autonomy**					0.0000
	Autonomous	456 (26.1)	119 (12.9)	575 (21.5)	
	Less autonomous	1,290 (73.9)	807 (87.1)	2,097 (78.5)	
**Economic empowerment**					
Owns land	No	1,741 (79.2)	903 (82.3)	2,645 (80.3)	0.1384
	Yes	456 (20.8)	194 (17.7)	650 (19.7)	
Owns house	No	1,724 (78.5)	898 (81.6)	2,622 (79.5)	0.1829
	Yes	473 (21.5)	203 (18.4)	676 (20.5)	
**Attitudes towards domestic violence**					
	Positive (No)	1,357 (63.4)	617 (57.0)	1,974 (61.3)	0.0112
	Negative (Yes)	782 (36.6)	465 (43.0)	1,247 (38.7)	
**Region**					
	North Central	275 (12.5)	136 (12.4)	411 (12.5)	0.0000
	North East	422 (19.2)	225 (20.4)	647 (19.6)	
	North West	401 (18.3)	463 (41.9)	864 (26.2)	
	South East	282 (12.8)	93 (8.5)	376 (11.4)	
	South South	359 (16.3)	67 (6.1)	427 (12.9)	
	South West	459 (20.9)	118 (10.7)	577 (17.5)	
**Religion**		0.0000			
	Christianity	1,310 (59.8)	347 (31.6)	1,657 (50.4)	
	Islam	865 (39.4)	741 (67.5)	1,606 (48.8)	
	Other	18 (0.8)	10 (1.0)	28 (0.9)	
**Age at death**					
	12–19	594 (26.3)	210 (21.4)	804 (24.6)	0.0000
	20–29	721 (33.5)	430 (40.0)	1,151 (35.8)	
	30–39	551 (26.0)	300 (31.1)	851 (27.7)	
	40–49	288 (14.2)	84 (7.6)	372 (11.9)	

Note: Some numbers may not add to the total due to missing cases in those records

To understand the differences in characteristics of the women and contextualize the results, Table 4 presents the characteristics of the deceased sisters as reported by their surviving sisters. In general, and for most of the characteristics assessed, the North West region had worse outcomes than the rest of the regions. For example, of all the poor households identified in the sample, the North West region had the largest proportion at 54.6% including the largest proportion of all the communities with low levels of community women’s education.

**Table 4:** Percent distribution of deceased sisters identified by the direct sisterhood method by individual characteristics and region of residence, Nigeria Demographic and Health Survey 2013 (n = 3,302).

Characteristics	North Central	North East	North West	South East	South South	South West
**Wealth index**						
Poor	10.4	25.8	54.6	4.3	2.6	2.4
Middle	21.2	10.2	24.7	13.9	17.6	12.5
Rich	11.7	5.4	12.2	15.2	20.5	35.1
**Residence**						
Urban	6.7	11.7	16.6	17.8	13.3	33.9
Rural	16.5	25.2	33.0	6.8	12.3	5.8
**Community women’s education**						
Low	8.4	33.2	55.3	0.9	0.0	2.1
Medium	19.6	19.5	14.3	14.8	14.4	17.5
Higher	10.5	5.2	5.4	19.6	25.4	33.9
**Highest educational attainment**						
No schooling	10.3	32.1	49.5	3.6	2.2	2.4
Primary	16.0	13.3	15.4	17.2	18.5	19.6
Secondary	11.8	9.7	7.4	16.9	23.0	31.2
Higher	15.9	10.8	8.2	14.2	14.5	36.4
**Spousal education difference**						
Equal	8.5	23.5	35.8	8.6	11.4	12.3
Wife higher	9.8	14.9	15.9	22.8	10.6	26.1
Husband/partner higher	19.3	20.0	20.3	8.9	13.2	18.5
**Autonomy**						
Autonomous	14.5	11.9	14.0	16.3	25.2	18.1
Less autonomous	11.3	24.0	33.1	8.4	7.1	16.1
**Economic empowerment**						
Owns land						
No	11.3	22.8	27.7	9.6	11.0	17.6
Yes	17.2	6.5	19.5	18.8	21.0	17.0
Owns house						
No	9.1	23.2	28.8	9.0	11.3	18.6
Yes	25.2	5.7	15.8	20.9	19.3	13.1
**Attitudes towards domestic violence**						
Positive (No)	12.9	15.6	24.1	9.3	13.9	24.3
Negative (Yes)	12.0	24.7	30.4	15.0	10.9	7.0
**Religion**						
Christianity	15.4	8.7	3.9	22.2	25.1	24.8
Islam	9.0	31.2	49.1	0.0	0.1	10.5
Other	37.4	9.6	10.5	34.0	7.0	1.5
**Age at death**						
12–19	11.5	20.5	30.5	12.7	12.0	12.9
20–29	13.8	23.1	24.0	12.4	13.0	13.7
30–39	14.2	19.5	20.3	14.8	13.3	18.0
40–49	10.7	18.1	21.2	17.3	14.4	18.4
Mean age at death (SD)*	27.9 (9.1)	25.7 (9.0)	24.5 (9.0)	28.3 (9.4)	27.3 (9.3)	27.8 (9.8)
**Total**	**12.5**	**19.6**	**26.2**	**11.4**	**12.9**	**17.5**
**Number of women**	**411**	**647**	**864**	**376**	**427**	**577**

Note

*Standard deviation. All differences were statistically significant.

### Multivariate analysis of pregnancy-related deaths on socio-demographic characteristics

Multilevel logistic regression results are presented in Table 5. Unadjusted ORs show that pregnancy-related deaths were less likely to be reported by sisters from households in the middle wealth index category than from households in the poor category [OR: 0.76; 95% C.I: 0.61–0.95]. Maternal deaths were also less likely to be reported by sisters from rich households compared with sisters from poor households [OR: 0.60; 95% C.I: 0.45–0.78]. Sisters with at least primary schooling were less likely to report deaths from pregnancy-related complications compared with sisters with no formal schooling (ORs ranging from 0.47 to 0.66). If a sister had more years of schooling than her husband/partner, she was less likely to report maternal death (OR: 0.59; 95% CI: 0.44–0.79) than when they had the same years of schooling. Similarly, if the husband/partner had more years of schooling than the wife, reporting of maternal deaths reduced by 19% (OR: 0.81; 95% CI: 0.65–0.99). Less autonomous sisters were 36% more likely to report deaths from maternal causes than autonomous women (OR: 1.06; 95% CI: 1.06–0.73). Practicing Islam was associated with odds of reporting maternal deaths that were roughly twice (OR: 1.82; 95% C.I: 1.44–2.31) those practicing Christianity. Pregnancy-related deaths were also more likely to be reported by women aged 20–29 and 30–39 than among the younger (OR: 2.07; 95% C.I: 1.65–2.58) and older age groups (OR: 2.07; 95% C.I: 1.63–2.63).

**Table 5:** Odds ratios and 95% confidence intervals for multilevel logistic regression models of maternal mortality on selected characteristics, Nigeria Demographic and Health Survey 2013.

Variable		Unadjusted odds ratios	Model 0 (Empty model)	Model 1 (Individual level variables)	Model 2 (Community-level variables)
**Fixed effects**					
***Individual characteristics***					
**Wealth index**	Poor (r)	1.00		1.00	1.00
	Middle	0.76 [0.61–0.95]*		0.78 [0.60–1.00]	0.91 [0.69–1.19]
	Rich	0.60 [0.45–0.78]***		0.63 [0.45–0.88]**	0.80 [0.53–1.20]
**Highest educational** **Attainment**	No schooling (r)	1.00		1.00	1.00
	Primary	0.66 [0.52–0.85]**		0.79 [0.59–1.05]	0.95 [0.71–1.29]
	Secondary	0.61 [0.50–0.79]***		0.71 [0.50–1.00]	0.92 [0.64–1.33]
	Higher	0.47 [0.33–0.68]***		1.17 [0.51–2.68]	1.28 [0.56–2.93]
**Spousal educational** **Difference**	Equal (r)	1.00		1.00	1.00
	Wife higher	0.59 [0.44–0.79]***		0.89 [0.62–1.28]	0.84 [0.59–1.21]
	Husband higher	0.81 [0.66–0.99]*		0.98 [0.78–1.23]	1.03 [0.82–1.30]
**Autonomy**	Autonomous (r)	1.00		1.00	1.00
	Less autonomous	1.36 [1.06–1.73]*		1.23 [0.95–1.60]	1.21 [0.93–1.58]
**Economic empowerment**					
Owns land	No (r)	1.00		1.00	1.00
	Yes	0.82 [0.66–1.03]		0.87 [0.64–1.16]	0.86 [0.64–1.15]
Owns house	No (r)	1.00		1.00	1.00
	Yes	0.81 [0.65–1.01]		1.09 [0.81–1.46]	1.13 [0.84–1.53]
**Attitudes towards domestic violence**	No (r)	1.00		1.00	1.00
	Yes	1.03 [0.86–1.24]		0.89 [0.73–1.09]	0.91 [0.74–1.12]
**Religion**	Christianity (r)	1.00		1.00	1.00
	Islam	1.82 [1.44–2.31]***		2.31 [1.77–3.00]***	1.52 [1.10–2.11]**
**Age at death**	12–19 (r)	1.00		1.00	1.00
	20–29	2.06 [1.65–2.58]***		2.00 [1.56–2.58]***	2.07 [1.61–2.66]***
	30–39	2.07 [1.63–2.63]***		1.88 [1.44–2.48]***	1.96 [1.49–2.57]
	40–49	1.07 [0.77–1.47]		0.94 [0.65–1.35]	0.98 [0.68–1.41]
**Residence**	Urban (r)	1.00			1.00
	Rural	1.61 [1.29–2.00]***			0.84 [0.63–1.12]
**Community women’s education**	Low (r)	1.00			1.00
	Medium	0.52 [0.39–0.68]***			0.67 [0.48–0.95]*
	High	0.37 [0.27–0.52]***			0.65 [0.40–1.05]
**Region**	North Central (r)	1.00			1.00
	North East	1.36 [0.87–2.13]			1.11 [0.75–1.64]
	North West	3.13 [2.03–4.83]***			2.14 [1.42–3.23]***
	South East	0.80 [0.49–1.32]			1.23 [0.76–1.98]
	South South	0.53 [0.32–0.86]*			0.68 [0.42–1.10]
	South West	0.61 [0.37–0.99]*			0.84 [0.54–1.31]
**Random effects**					
*Community-level*					
Variance (SE)			0.810 (0.136)***	0.326 (0.109)**	0.269 (0.103)**
Intracommunity correlation (SE)			0.197 (0.027)	0.090 (0.028)	0.076 (0.027)
Number of observations		3,302	3,302	2,343	2,343
LR test vs. logistic model			102.40	15.18	10.95
Prob > Chi2		0.000	0.000	0.000	0.001

Notes

SE–Standard error

***p<0.001

**p<0.01

*p<0.05

The number of cases reduced between Models 0, 1, and 2 due to some missing variables across some variables.

In Table 5, results also show that sisters in rural areas were 61% more likely to report maternal deaths (OR: 1.61; 95% C.I: 1.29–2.00) than sisters in urban areas. Sisters living in communities with medium and high levels of community women’s education were associated with lower odds of maternal deaths [OR: 0.52; 95% C.I: 0.39–0.68] and [OR: 0.37; 95% C.I: 0.27–0.52], respectively, than sisters in communities with low levels of community women’s education. The odds of reporting maternal deaths more than tripled among sisters in the North West region (OR: 3.13; 95% C.I: 2.03–4.83) than in the North Central region, and the odds were lower in the South-South (OR: 0.54; 95% C.I: 0.32–0.86) and the South West (OR: 0.61; 95% C.I: 0.37–0.99) than in the North Central region. There was no statistically significant effect of economic empowerment variables and attitude towards domestic violence on reporting of maternal deaths, however, these variables have been retained in the multivariable model due to their theoretical importance in the association with maternal mortality.

Table 5 also presents the multi-level regression results for the empty model (Model 0), the individual-level (Model 1) and the full Model 2 (with community-level variables). The total variance in pregnancy-related mortality associated with community context was initially estimated using the empty model. The variance was significant across communities (*τ* = 0.810, p = 0.000). The intra-community correlation was 19.7% indicating that there was variance in pregnancy-related mortality at the community level. A likelihood ratio (LR) test comparing the empty model to an ordinary logistic regression model was highly significant for the data. Wealth index, highest educational attainment, spousal educational difference, autonomy, economic empowerment, attitudes towards domestic violence, age at death, and religion were introduced in Model 1 as individual-level factors. When compared to the crude ORs in Table 5, results in Model 1 showed that the following variables remained significant in the adjusted Model 1: women from rich households (OR = 0.63; 95% C.I: 0.45–0.88); women practicing Islam (OR = 2.31; 95% C.I: 1.77–3.00); women aged 20–29 years (OR = 2.00; 95% C.I: 1.56–2.58); and women aged 30–39 years (OR = 1.88; 95% C.I: 1.44–2.48).

In comparison to the empty model, the variation in pregnancy-related mortality remained significant across women (*τ* = 0.326, p = 0.003). The intra-community correlation was 9.0% indicating that there was variance in pregnancy-related mortality at the community level. An LR test comparing Model 1 to an ordinary logistic regression model was highly significant for the data. With the introduction of residence, community women’s education, and region in Model 2, the likelihood of a woman dying of pregnancy-related causes was higher among Muslim women (OR = 1.52; 95% C.I: 1.10–2.11) and among women aged 20–29 years (OR = 2.07; 95% C.I: 1.61–2.66). Women living in areas with medium level of community women’s education were 33% more likely to die from pregnancy-related causes than women living in areas with low levels of community women’s education (OR = 0.67; 95% C.I: 0.48–0.95). By region of residence, women in the North West region were 2.14 times more likely to die from pregnancy-related causes than women from the North Central region (OR = 2.14; 95% C.I: 1.42–3.23). In comparison to Model 1, the variation in pregnancy-related mortality also remained significant across women (*τ* = 0.269, p = 0.009). The intra-community correlation was 7.6% indicating that there was variance in pregnancy-related mortality at the community level. An LR test comparing Model 1 to an ordinary logistic regression model was highly significant for the data.

In the regression models, we tested interaction effects for selected variables such as religion and region; including other autonomy variables. None of the interactions were significant and they did not improve the model fit. The overall results also show that the effect of the crude ORs attenuated in the adjusted model implying the mediating effect of the variables considered in the model in influencing pregnancy-related mortality.

**Fig 1:**
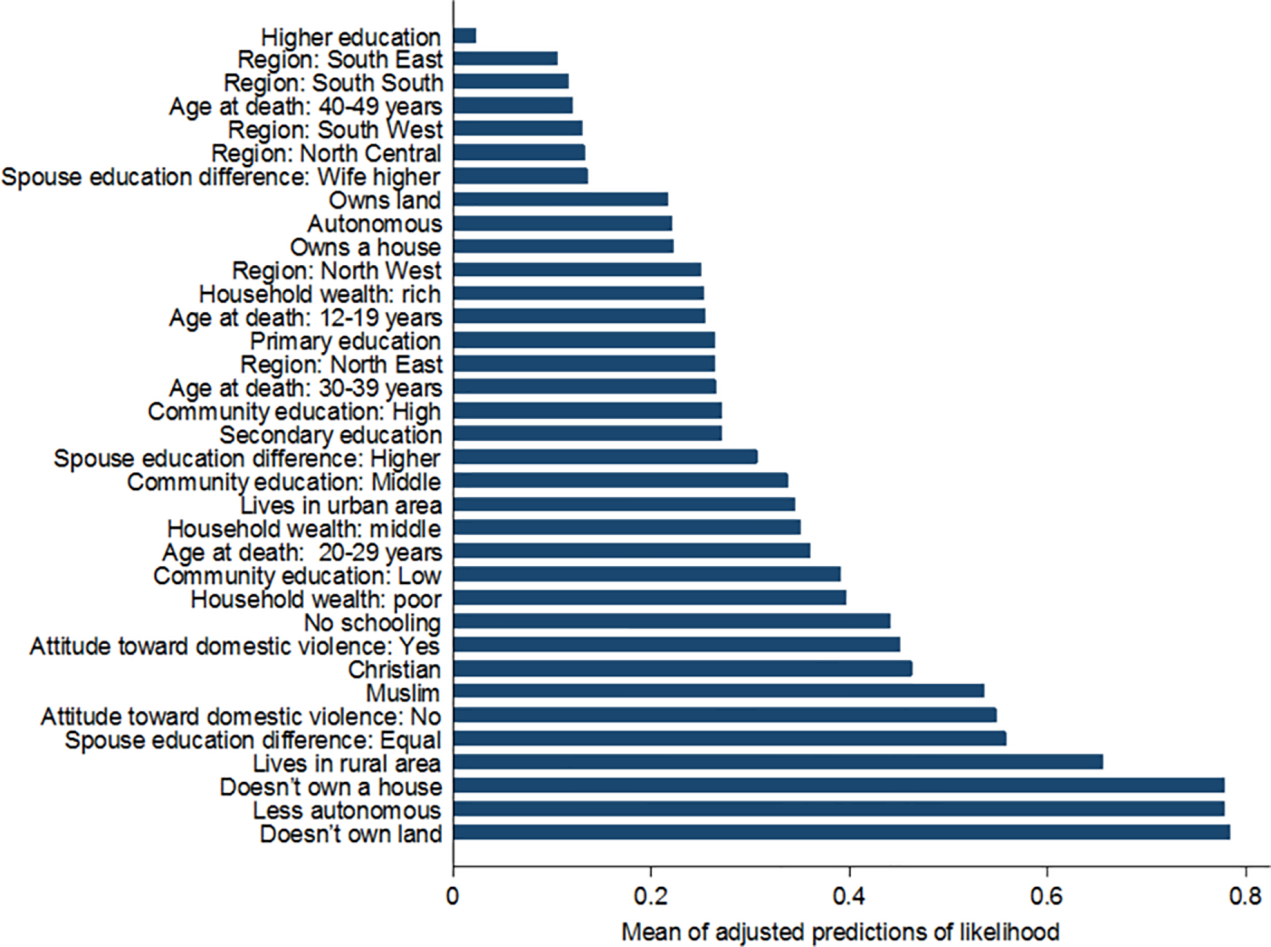
Adjusted predictions of the likelihood (odds ratios) of pregnancy-related mortality at the means of the independent variables, Nigeria Demographic and Health Survey 2013.

The adjusted prediction of likelihood model (Fig 1) depicts the likelihood of sisters reporting maternal deaths at the means of the independent variables using the estimates provided in Model 2 of Table 5. If all the other variables are set at their means or average values, then the predicted likelihood of sisters reporting pregnancy-related deaths was highest among sisters who did not own land followed by less autonomous sisters, sisters who did not own a house, and sisters residing in rural areas. These results underscore the importance of considering the variations in individual-level factors and community-level factors in explaining pregnancy-related mortality. In addition, these results are important in identifying key areas for intervention as the main determining factors for influencing pregnancy-related mortality when the other variables are held at their means or average values. Thus, the difference between the highest and lowest determines the extent to which gaps exist in areas of focus to reduce pregnancy-related mortality.

We compared results from the observed pregnancy-related mortality with similar results from the post-estimation multilevel regression models to assess the extent of variation in the observed and predicted pregnancy-related mortality. Results showed that in the adjusted model, the observed pregnancy-related mortality was almost 7% lower than the expected pregnancy-related mortality (i.e. 31.3% vs 33.6%) (Table 6). Across all socio-demographic characteristics, the observed pregnancy-related mortality was 3% higher than expected among reporting sisters living in the North Central region and among sisters with secondary schooling (Ratio = 1.03); with the South South region registering the highest observed pregnancy higher than expected at 20% (Ratio = 1.20) (Table 6).

**Table 6:** Observed and expected proportion of pregnancy-related deaths by selected characteristics identified by the direct sisterhood method.

Characteristics		Pregnancy-related deaths		Ratio (3) = (1) / (2)
		Observed (1)	Expected (2)	
**Age at death**				
12–19		26.1	28.0	0.93
20–29		37.4	39.8	0.94
30–39		35.3	35.9	0.98
40–49		22.6	22.4	1.01
**Wealth index**				
Poor		41.1	43.0	0.98
Middle		29.0	29.9	0.97
Rich		21.8	23.7	0.92
**Region**				
North Central		28.5	27.7	1.03
North East		32.2	34.7	0.93
North West		53.3	54.7	0.97
South East		24.2	25.3	0.96
South South		17.4	14.5	1.20
South West		19.4	21.2	0.91
**Religion**				
Christianity		20.9	20.9	1.00
Islam		43.5	44.4	0.98
**Residence**				
Urban		26.3	29.2	0.90
Rural		34.5	35.9	0.96
**Community women’s education**				
Low		47.0	47.5	0.99
Middle		27.5	13.2	1.00
Higher		19.9	20.8	0.96
**Highest educational attainment**				
No schooling		44.6	45.0	0.99
Primary		26.1	26.8	0.97
Secondary		22.8	22.1	1.03
Higher		19.3	25.7	0.75
**Spousal educational difference**				
Equal		35.9	36.8	0.97
Wife higher		24.1	25.1	0.96
Husband/partner higher		30.7	31.5	0.97
**Autonomy**				
Autonomous		23.0	23.6	0.97
Less autonomous		35.3	36.5	0.97
**Economic empowerment**				
Owns land	No	32.3	35.2	0.92
	Yes	26.5	27.8	0.95
Owns house	No	32.6	35.1	0.93
	Yes	26.4	28.4	0.93
**Attitudes towards domestic violence**				
Positive (No)		30.1	32.9	0.91
Negative (Yes)		33.1	35.0	0.95
**All deaths**		**31.3**	**33.6**	**0.93**

## Discussion

Our investigation on the sociocultural correlates of pregnancy-related mortality in Nigeria found a significant variation across communities. There was a significant association between pregnancy-related mortality and religion, age, community women’s education and region of residence. The observed association between community-level factors with maternal mortality is congruent with findings from previous studies [8,9,11,14,15]. Women in Northern Nigeria have historically experienced higher rates of maternal mortality compared to women in the Southern parts of the country [31]. Maternal mortality ratios of up to 1,012 deaths per 100,000 live births, one of the highest estimates globally, have been reported in Jigawa state in Northwestern Nigeria [32].

After adjusting for age and other sociocultural factors, region of residence remained a significant predictor of reporting maternal mortality; this suggests the existence of additional factors in Northwestern Nigeria which puts women at risk for maternal mortality. In his review, Lewis Wall describes the confluence of determinants that propagate maternal mortality in the region[15]. These determinants include a paucity of facilities to deal with obstetric emergencies, harmful traditional medical beliefs and practices, a political culture of rampant corruption and inefficiency, a deteriorating economy, strict male control and restriction of women’s access to medical care [15]. As such, many women in Northern Nigeria are less likely to report utilizing antenatal care, delivery in a healthcare facility, and the presence of skilled birth attendants during delivery [6,10].

While this study did not include measures of religiosity to assess the specific attributes or intensity of religious affiliation, such as the number of times people pray or go to prayer meetings. It is important to note that, Islam is the predominant religion of practice in northern Nigeria. Religious tenets which uphold women’s obedience to their partners or husbands, acceptance of strict confinement to the compound (purdah) or requirement of partner/husband’s permission to leave the confines of the house are likely to inhibit women from independently deciding to access health services or calling for timely assistance in the event of maternal complications [33,34].

Although there is a paucity of studies linking the level of community women’s education and maternal mortality, the level of community women’s education has also been associated with an increased likelihood of delivering a baby in a health facility in other studies [35,36]. A previous study on the determinants of maternal health care utilization found that Nigerian communities with a high proportion of women with secondary or higher education had four times the odds of delivering in a health facility [35]. This suggests that efforts to increase women’s educational attainment in communities with high maternal mortality ratios are necessary to accelerate the uptake of health services, which are crucial in the prevention of maternal mortality.

In line with Sustainable Development Goals, striving for gender equality (SDG-5) across all spheres and improving women’s agency should be at the core of strategies to improve maternal health [37]. Enabling women to make decisions for themselves has far-reaching implications for their health, the health of their families and communities. Utilizing a life-course approach to impact the health, education, and agency of Nigerian women, from childhood, through adolescence and adulthood will significantly contribute to achieving gender equality. Implementing this approach will require interventions across the socioecological system, starting from revisions in policies on child marriage, and gender parity and equality, investments in women’s education, to the provision of quality health services and infrastructure.

Programmatic implications of the study findings indicate that for an ethnically diverse country such as Nigeria, one-size-fits-all programs cannot be effective in reducing maternal mortality. Therefore, effective dissemination of evidence-based interventions requires considering the contextual environment in which women reside. Intervention programs should partner with community and religious groups to identify barriers to the education of the girl-child and traditional and religious beliefs associated with childbirth [38]. This strategy would provide leverage points to identify blind-spots and implement relevant and effective programs. These programs should also include community-based strategies such as training of midwives within religious and community groups, and disseminating reproductive and obstetric health information through women’s groups. Community engagement plays an important role in creating multiple avenues for women to freely discuss their health concerns [39]. This ensures that women have a respected role in preserving the expectations regarding their gender norms and at the same time seeking ways to improve their health [39].

Despite the strength and methodological quality of the DHS data in providing readily available data to estimate maternal mortality in low and middle-income countries [3,40], these findings should be interpreted in light of several limitations. The DHS surveys collect data on pregnancy-related mortality through inquiries about the deceased from their surviving sister, therefore the data cannot be used to make direct inferences about predictors of maternal mortality. The method of estimating the predictors of maternal mortality by proxy using sibling characteristics introduces the potential for misclassification of exposure status, as the reported cultural and social indices are experiences of the surviving sibling not necessarily those of the fatality case. However, existing evidence shows that the sociodemographic characteristics of the deceased sister is often similar to that of the reporting sister, thus confirming the appropriateness of using the reporting sister’s characteristics as proxy variables for the deceased sister [17,41].

Due to data limitations, adjustment for potential confounders in this study is likely incomplete. However, the aim of our study is not to examine causal effects but to provide suggestions for potential correlations that can be further assessed using cohort studies. Respondents were also not asked the marital status of the siblings. In a society, such as Nigeria, where pregnancies out-of-wedlock are more socially and culturally unacceptable, there is a chance for underestimation of abortion-related deaths as siblings may have been unaware that their sisters were pregnant. Additionally, underestimation of maternal mortality could have occurred, as deaths with unknown pregnancy status were categorized as non-maternal deaths. In addition, due to the fragile safety in the northern regions at the time of data collection, some clusters could not be assessed as such the data may not be entirely representative in those states [4].

Lastly, the weakness of the measurement variables for women’s empowerment (economic empowerment, autonomy in decision making and attitudes towards domestic violence) limits the study analysis and findings. Variables which accurately measure indices of women’s empowerment, both qualitatively and quantitatively, are necessary to adequately investigate their association with maternal mortality [42,43]. However, there are currently no cross-culturally validated measures to assess these constructs [14,42,44]. Future studies can build on existing research to develop and empirically evaluate measures and theoretical models which assess and further investigate the association between women’s empowerment and maternal health status and outcomes. This work would be important to develop and disseminate contextual, culturally-relevant and effective interventions to reduce maternal mortality [39].

In conclusion, our findings show little improvement from studies, reported more than a decade ago, on the sociocultural correlates of maternal mortality, particularly among women in Northwestern Nigeria [15,45]. Implementation of the SDG agenda to reduce maternal mortality needs to acknowledge the protracted nature of challenges facing women in poor and resource-constrained parts of Nigeria and the importance of engaging all levels across the socioecological system in marshalling resources, long-term support and implementing strategies which ensures that by 2030, each woman has an improved lifetime risk of maternal mortality and a better status in life.
